# Recombinant Production and Characterization of a Novel α-L-Fucosidase from *Bifidobacterium castoris*

**DOI:** 10.3390/ijms26199344

**Published:** 2025-09-24

**Authors:** Burcu Pekdemir, Sercan Karav

**Affiliations:** Department of Molecular Biology and Genetics, Çanakkale Onsekiz Mart University, 17100 Çanakkale, Turkey; burcupekdemir0@gmail.com

**Keywords:** α-L-fucosidase, L-fucose, defucosylation, recombinant enzyme production

## Abstract

α-L-fucosidases (EC 3.2.1.51) are of particular interest due to their ability to cleave terminal α-L-fucose residues from glycoconjugates, a property associated with numerous biological and therapeutic effects. They have also been investigated for their potential use in glycan remodeling, disease biomarker analysis, and particularly as therapeutic agents in the context of fucosidosis, a rare lysosomal storage disorder, caused by a deficiency in α-L-fucosidase activity. However, limitations in enzyme availability, stability, and substrate specificity highlight the need for novel and more efficient enzyme sources. *Bifidobacterium castoris* (*B. castor* is) is a newly identified species first discovered in the beaver gut microbiota in 2019. Phylogenetic studies have revealed its advanced metabolic capacity, and genomic analyses have demonstrated its extensive carbohydrate metabolism potential. This research article focuses on the recombinant production and biochemical characterization of a novel α-L-fucosidase from *B. castoris* LMG (Laboratorium voor Microbiologie Gent) 30937, predicted to belong to glycoside hydrolase family 29 (GH29) according to Universal Protein Resource (UniProt) annotation. Under optimized reaction conditions the recombinant α-L-fucosidase exhibited a specific activity of 0.264 U/mg to pNP-Fuc (4-Nitrophenyl-α-L-fucopyranoside). The results indicate that the enzyme is active in the pH range of 3.0–8.0 and temperatures of 24–42 °C, but its optimum conditions are the slightly acidic pH of 5.5 and the elevated temperature of 42 °C. This profile suggests that the enzyme is adapted to acidic intestinal-like environments. This novel enzyme expands the GH29 α-L-fucosidase repertoire and offers a promising new candidate for future biotechnological applications.

## 1. Introduction

α-L-fucosidases (EC 3.2.1.51) are enzymes that hydrolyze fucosyl bonds or catalyze the transfer of fucosyl residues by acting on oligosaccharide and glycosphingolipid substrates resulting from modifications in glycosylation and proteolytic processing. Among these substrates, fucose-containing oligosaccharides, classified as glycans formed by the assembly of several oligosaccharide units into complex structures, play critical roles in various biological processes [1]. The ABO blood group and Lewis antigens are examples of oligosaccharides bearing fucosyl groups and are important in biology [2]. Changes in the expression of antigens in the ABO system can affect an organism’s susceptibility to various infections [3]. Changes in the expression of ABO and Lewis antigens are associated with prognosis in some types of cancer [4,5,6]. Furthermore, fucosylated oligosaccharides play critical roles in fertilization and early embryogenesis [7,8]. Brain development is a key organ in this process, and functions related to learning and memory, such as neurite outgrowth, migration, and synapse formation, are regulated by fucosylated oligosaccharides [9,10]. Although numerous studies have demonstrated the important functions of fucose-linked oligosaccharides in biological systems, more detailed investigations are needed to fully elucidate the underlying mechanisms of these molecules. In particular, the discovery of highly active α-L-fucosidases and their comprehensive enzymatic characterization are crucial for better understanding the structural and functional properties of fucose. According to the Carbohydrate Active Enzymes database (CAZy database), five GH families (GH139, GH141, GH151, GH95, GH29) encoding α-L-fucosidase have been identified [11]. α-L-fucosidases use one of two mechanisms, a dual replacement retention and reversal mechanism, to catalyze the hydrolysis of terminal α-fucosidic linkages. Fucosidases using the dual replacement retention mechanism belong to the GH29 family and have extensive substrate specificity; belonging to the GH95 family, which use the reversal mechanism, are insufficient in targeting. Compared to the GH29 family, GH95 fucosidases are less characterized. Additionally, GH95 α-L-fucosidases are found in bacteria, fungi and plants, but not in animals [12]. GH139, GH141 and GH151 fucosidases belong to relatively newly established families, and their catalytic mechanisms remain to be demonstrated experimentally [12,13]. According to the CAZy database, approximately 9,867 α-L-fucosidase sequences belonging to the GH29 family have been identified, with 96% of them being of bacterial origin. The GH95 family is the second most abundant group, comprising around 4,890 sequences, 97% of which are also derived from bacteria. Smaller families such as GH139, GH141, and GH151 are likewise predominantly bacterial in origin. Taken together, these data indicate that approximately 96.5% of all known fucosidase sequences are of bacterial origin. Additionally, it has been reported that some bacterial genomes contain a high number of GH29 and GH95 genes in multiple copies [11,14].

Microbial enzymes, which offer advantages over plant- and animal-derived enzymes, have become dominant in the commercial enzyme market due to their higher production yields, easier optimization and genetic manipulation, and economical production processes. Although microbial enzymes constitute 88% of commercial enzymes, their numbers are quite limited. Therefore, the discovery of microbial enzyme sources is crucial [15]. Our increasing understanding of the biological roles of microbial α-L-fucosidases, resulting from biochemical and structural analyses, has highlighted the importance of metagenome studies. Recent metagenomic analyses of fecal samples from breastfed infants have revealed a notably high abundance of GH29 family α-L-fucosidases in the infant gut environment. This diversity of α-L-fucosidases may confer a colonization advantage to microbes in both infant and adult intestines. Furthermore, the ability to remove α-L-fucosyl residues from free oligosaccharides and glycoconjugates provides fucosidase-producing microbes with a competitive edge in mucus glycan foraging, which in turn contributes to the maintenance of intestinal homeostasis. Although metagenomic databases contain extensive sequencing data, studies on the structure-function relationships of GH29 α-L-fucosidases remain limited [11]. Therefore, to address the existing knowledge gaps regarding the biological roles of α-L-fucosidases in the literature, a combination of metagenomic and glycomic approaches is needed. The structural and functional diversity of microbial α-L-fucosidases represents a valuable resource for glycan analysis, biomarker discovery, and the development of innovative glycan-targeted therapeutic strategies. Our study contributes to addressing existing gaps in knowledge regarding the biological functions of α-L-fucosidases by demonstrating the enzyme’s substrate specificity and activity profile. These findings provide a strong foundation for research to more comprehensively understand the ecological and biological functions of α-L-fucosidases in gut-associated niches, where their roles in host-microbe interactions are poorly understood. Enzymes produced recombinantly from microbial sources are also attracting significant interest in enzyme replacement therapy (ERT) due to their advantages over plant- and animal-derived enzymes. The potential use of these enzymes as therapeutic agents offers a promising strategy for disease treatment. Enzyme replacement therapy is a widely used approach, particularly in cases of enzyme deficiency or deficiency in lysosomal storage diseases. This treatment relies on the replacement of an enzyme that is not naturally produced in sufficient quantities by exogenous administration at regular intervals. From an α-L-fucosidase perspective, fucosidosis, a rare autosomal recessive disease characterized by accumulation of fucose-linked glycoconjugates in the lysosomes, is a lysosomal storage disease. This rare, autosomal recessive disease is characterized by rapid neurodegeneration. Due to its rarity and the fact that patients typically die at an early age, there is currently no effective treatment. Efficient bacterial α-L-fucosidases that are easily manipulated, highly active, and do not produce undesirable byproducts are required for ERT treatment studies in fucosidosis [16]. In conclusion, α-L-fucosidases offer a broad range of applications in fields such as glycan engineering, biotechnology, and disease monitoring, making their discovery crucial for advancing biotechnological progress [11].

This research article focuses on the recombinant production and characterization of α-L-fucosidase from *B. castoris* LMG 30937, classified under the GH29 family, as predicted by the Universal Protein Resource (UniProt) annotation. *B. castoris* which was isolated from beaver microflora for the first time in 2019, was detected in the microflora of wild mice in another study in 2022. It contributes the limited knowledge of the metabolic capabilities of wild animal populations. It has been reported to be rich in glycosidases, which especially degrade fucose and other complex sugar structures. *B. castoris* has ~86 CAZymes, and 25 GH families have been identified in this species. Among these, there are the GH95 and GH29 families, which encode the α-L-fucosidase [17,18]. While the corresponding gene belonging to the GH29 family has been previously annotated in genomic databases using automated computational predictions based on protein homology, this study demonstrates the enzyme’s functional expression and biochemical activity. This experimental validation is particularly significant, as many annotated glycoside hydrolases remain uncharacterized, and computational predictions alone cannot reveal substrate specificity, kinetic properties or physiological roles. This distinction highlights the importance of our study. While computational annotations suggest the existence of GH29 α-L-fucosidase, substrate specificity, kinetic potential, and ecological relevance can only be revealed through recombinant production and biochemical analyses. Furthermore, biochemically characterized GH29 α-L-fucosidase was shown to exhibit activity in the pH range of 3.0–8.0 and temperatures of 24–42 °C. Optimum conditions were determined as a slightly acidic pH of 5.5 and a high temperature of 42 °C. This profile suggests that the enzyme is adapted to acidic intestinal-like environments. This novel enzyme expands the repertoire of GH29 α-L-fucosidase and offers a promising candidate for future biotechnological applications. Future studies can further investigate its substrate coverage, kinetic parameters, and stability under industrial processing conditions.

## 2. Results

### 2.1. Cloning of α-L-Fucosidase

**Figure 1:**
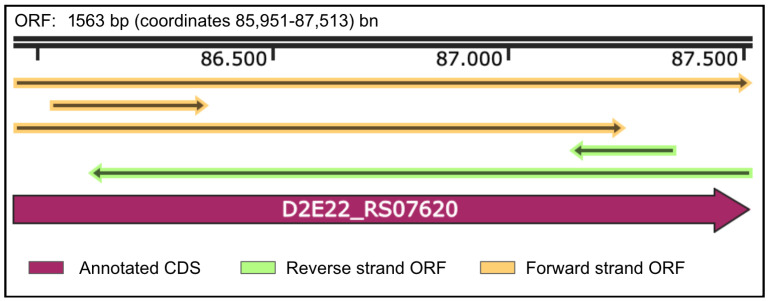
Gene sequence diagrams of the B. castoris GH29 α-L-fucosidase. The gene (ORF: 1563 bp, 85,951–87,513) encodes a protein of 520 amino acids, with locus tag D2E22_RS07620 and accession number NZ_QXGI01000006.1.

**Figure 2:**
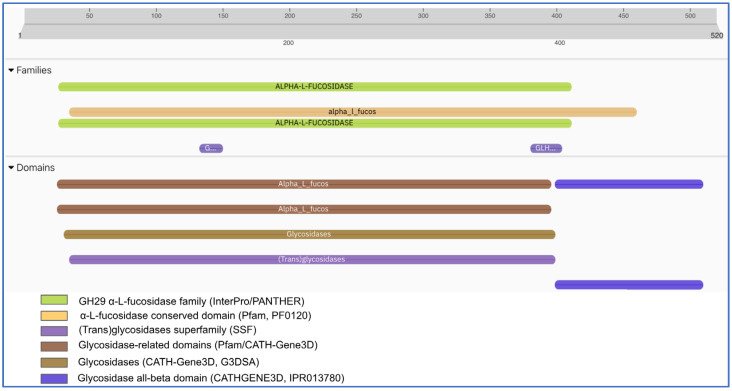
Protein sequence diagrams of the B. castoris GH29 α-L-fucosidase. SignalP/TMHMM indicate no signal peptide/transmembrane helices. Functional domains were identified by multiple databases. Conserved domain and family analyses confirm that the protein belongs to the GH29 α-L-fucosidase family (IPR000933). Accession: WP_241218324.1; encodes protein: 520 aa. (Data from NCBI GenBank and NCBI CDD; schematic generated using the InterPro (106.0)) and SnapGene (5.2.4) software.

The gene and protein features of the *B. castoris* α-L-fucosidase are summarized in Figure 1 and Figure 2. The ORF (1563 bp; coordinates 85,951–87,513) encodes a 520 amino acid protein (locus tag D2E22_RS07620; accession NZ_QXGI01000006.1). Domain analyses revealed that the protein belongs to the GH29 α-L-fucosidase family (IPR000933), with conserved α-L-fucosidase motifs (PF0120). In addition, the enzyme clustered within broader glycosidase/transglycosidase superfamilies (CATH-Gene3D, SSF), consistent with its evolutionary relationship to other glycoside hydrolases. No signal peptide or transmembrane helices were predicted, suggesting a cytoplasmic localization. These results confirm the gene’s functional annotation and highlight its role as a member of the GH29 α-L-fucosidase family.

An α-L-fucosidase gene was amplified from the genomic DNA of *B. castoris* LMG 30937 using designed primers which contain an N-terminal 6xHis-SUMO (Small Ubiquitin-like Modifier) tag. After transformation of PCR-amplified gene and the pRham™ N-His SUMO vector into *E. cloni* 10G chemically competent cells, the transformed cells were screened by using the LB agar plate containing kanamycin (33 ug/mL). Colony PCR screening using sequenced primers, provided Expresso™ Rhamnose Cloning and Expression Kit, was conducted on four randomly selected colonies. All positive colonies produced amplification bands of the expected size (1563 bp), confirming the correct presence of the recombinant insert (Figure A2). DNA fragment with the expected molecular size was sequenced for final confirmation.

### 2.2. α-Fucosidase Production

**Figure 3:**
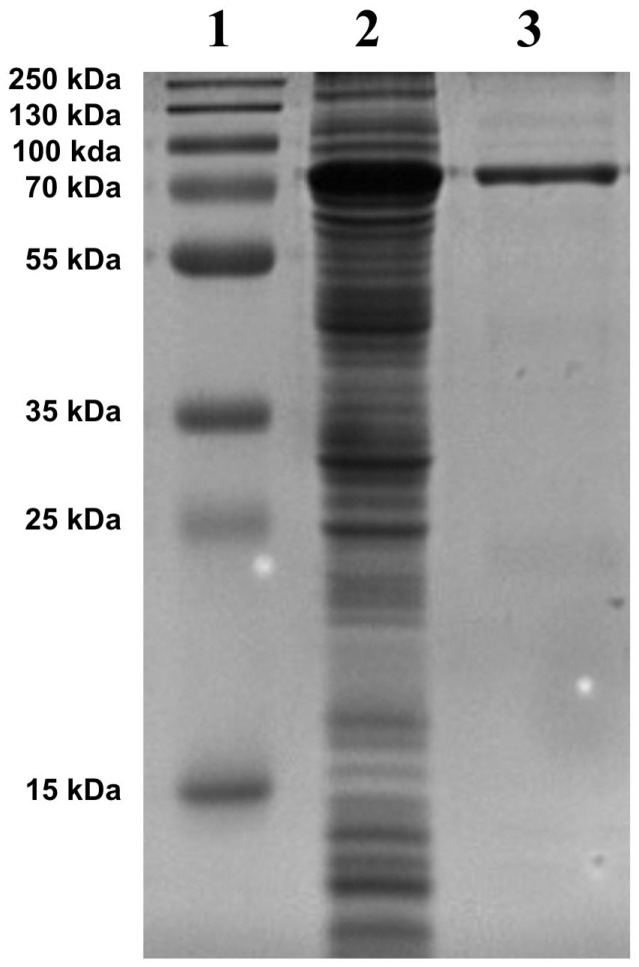
SDS-PAGE of α-L-fucosidase recombinantly produced in *E. cloni* 10G chemically competent cells. Sample was loaded onto 4–12% SDS-PAGE and gels were stained with Commassie Brilliant Blue. Lane 1, protein standard; Lane 2, crude enzyme; Lane 3, α-L-fucosidase (70.36 kDa).

A total of 1.02 mg of 6xHis SUMO tagged α-L-fucosidase was obtained from 50 mL of LB culture under optimized induction conditions (2% final concentration L-rhamnose, 24 °C for 16 h), corresponding to a yield of approximately 20.4 mg/L. The purity of the concentrated recombinant α-L-fucosidase was assessed by SDS-PAGE analysis (Figure 3). A single band was observed at approximately 70.36 kDa, which is consistent with the expected size of the enzyme fused to a SUMO and His-tag (~12 kDa total tag size). The Coomassie Brilliant Blue-stained gel showed no additional bands, indicating >95% purity.

### 2.3. Confirmation of Recombinantly Produced α-L-Fucosidase Activity and Determination of Optimum Reaction Conditions

**Figure 4:**
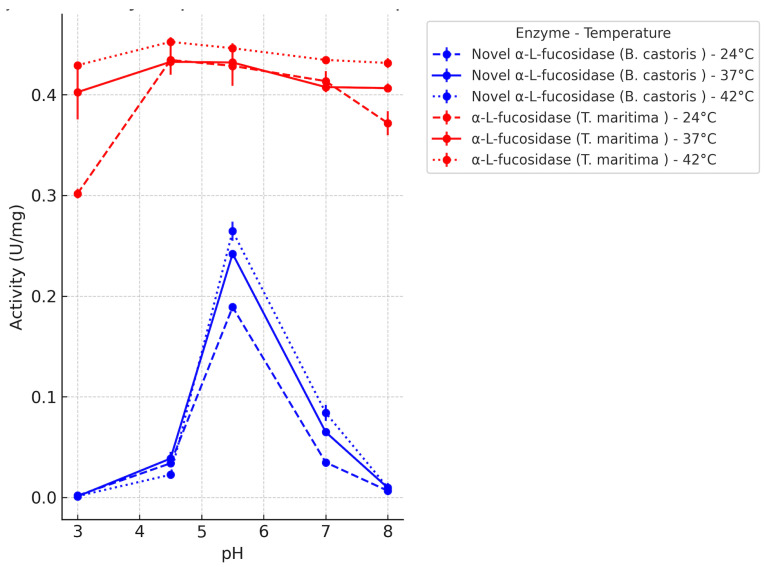
Enzymatic characteristics of α-L-fucosidases from *T. maritima* and *B. castoris* under different pH (3.0, 4.5, 5.5, 7.0, and 8.0) at three temperatures (24 °C, 37 °C, and 42 °C). Enzyme activities are presented as mean ± SD (*n* = 3).

The catalytic activity of the recombinant *B. castoris* α-L-fucosidase was systematically evaluated across a pH range of 3.0–8.0 and at three distinct temperatures (24 °C, 37 °C, and 42 °C) and subsequently benchmarked against the commercial *T. maritima* α-L-fucosidase under identical assay conditions. For each temperature, line graphs were plotted showing the mean activity values with standard deviations (Figure 4). The comparative activity profiles revealed distinct stability characteristics between the two enzymes. Under identical assay conditions, the recombinant *B. castoris* α-L-fucosidase exhibited a slightly lower maximal activity than the well-characterized commercial α-L-fucosidase from *T. maritima*.

**Figure 5:**
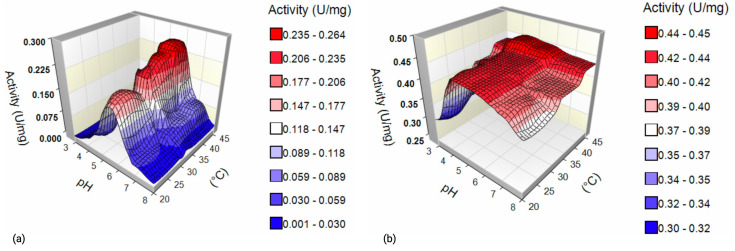
Comparative 3D surface plots of α-L-fucosidase activity as a function of pH and temperature. (**a**) *B. castoris* enzyme, showing greater sensitivity to pH variation. (**b**) *T. maritima* enzyme, showing greater sensitivity to temperature variation.

To further illustrate the effect of pH and temperature on enzymatic activity, 3D surface plots were created for each enzyme (Figure 5). As seen in Figure 5, the activity landscapes of the two α-L-fucosidases differ primarily in their sensitivity. The *B. castoris* enzyme maintained its maximum activity under slightly acidic conditions (pH 5.5) when the reaction temperature varied between 24 and 42 °C, suggesting that its performance is governed by pH rather than thermal fluctuations. In contrast, although the commercial *T. maritima* α-L-fucosidase showed a wide tolerance to pH changes (pH 3.0–8.0), it was strongly affected by temperature changes, and its optimum activity was observed under high thermal conditions. This contrast highlights a complementary adaptive strategy: the *B. castoris* enzyme is predominantly pH sensitive, while the *T. maritima* enzyme is predominantly temperature sensitive. These differences may influence their selection for biocatalytic processes where pH stability or thermal stability are required.

Enzyme activities are expressed as mean ± standard deviation (U/mg) calculated from triplicate measurements. Two-way analysis of variance (Two-way ANOVA) showed that both pH (F(4,30) = 4844.92, *p* < 0.0001) and temperature (F(2,30) = 132.16, *p* < 0.0001) had a significant effect on enzyme activity. A significant interaction between pH and temperature was also found (F(8,30) = 64.63, *p* < 0.0001). These findings suggest that the effect of temperature varies depending on the pH level. The highest mean activity was observed at pH 5.5 and 42 °C (0.264 U/mg).

Tukey–Kramer post hoc tests confirmed that pH 5.5 was the optimal pH at each temperature, as the enzyme activity at pH 5.5 was significantly greater than at any other pH within the same temperature (Tukey-adjusted *p* < 0.05 for all pairwise comparisons). The highest observed activity was at 42 °C and pH 5.5 (0.264 ± 0.004 U/mg), which was significantly higher than the next-highest condition (pH 5.5 at 37 °C, 0.242 ± 0.004 U/mg) as well as all other conditions. The *p*-value in each row in the table was obtained by comparing the corresponding pH with pH 5.5 (Table 1). These results demonstrate that the enzyme’s activity is maximized at pH 5.5 and is enhanced by higher temperature within the range tested, while extremely acidic or alkaline pH conditions dramatically reduce the activity (especially at lower temperatures).

## 3. Discussion

In this study, we successfully produced and characterized a recombinant α-L-fucosidase from *B. castoris* LMG 30937, classified within the glycoside hydrolase family 29 (GH29). It was isolated from beaver microflora for the first time in 2019 and detected in the microflora of wild mice in another study in 2022. It contributes to the limited body of knowledge regarding the metabolic capabilities of wild animal populations. It has been reported to be rich in glycosidases, which especially degrade fucose and other complex sugar structures. *B. castoris* has ~86 CAZymes, and 25 GH families have been identified in this species. Among these, there are the GH95 and GH29 families, which encode the α-L-fucosidase [17,18]. While the corresponding gene belonging to the GH29 family has been previously annotated in genomic databases using automated computational predictions based on protein homology (Figure 1 and Figure 2), this study demonstrates the enzyme’s functional expression and biochemical activity. Our findings demonstrate that the recombinant enzyme is functionally active against synthetic substrates such as pNP-α-L-fucose, thereby supporting the accuracy of its prior annotation and highlighting its potential role in fucose metabolism within the host environment (Figure 4).

The enzyme demonstrated a well-defined pH- and temperature-dependent activity profile, with maximum hydrolysis of pNP α L fucose at pH 5.5 and 42 °C (0.264 U/mg) and maintained appreciable catalytic activity across adjacent pH and temperature ranges (Figure 4). Statistically significant main effects of pH and temperature, as well as their interaction, were confirmed by two-way ANOVA followed by Tukey post hoc analysis (Table 1). These results underscore the enzyme’s adaptability to slightly acidic and elevated thermal environments, aligning with the gut associated niche of Bifidobacteria. This activity profile is consistent with reported features of GH29 α-L-fucosidases from other *Bifidobacterium* species that reside in the mammalian gut. For instance, the GH29 enzyme AfcB from *Bifidobacterium bifidum* shows optimum activity at pH 5.5–6.0 and 45 °C and remains stable across a broad pH range (pH 3.5–8.0) and temperatures below 50 °C [19,20,21]. These parallels reinforce the plausibility of the novel enzyme’s behavior as part of the bifidobacterial GH29 repertoire adapted to gastrointestinal conditions. Among the characterized GH29 α-L-fucosidases from Bifidobacterium, *B. bifidum* AfcB exhibits specificity for α1,3/α1,4 bonds and does not hydrolyze pNP-fucoside. This property is consistent with the B-subfamily of GH29, which is intolerant to pNP-fucoside [19]. The proposed framework for bifidobacterial GH29s predicts that GH29-A members are generally able to hydrolyze pNP-fucoside, while GH29-B members have a pronounced α1,3/4 bond specificity and show weak or no activity against pNP-Fuc. In this context, the stable activity of *B. castoris* enzyme on pNP-fucoside distinguishes it from AfcB and is consistent with a GH29-A-like behavior [20]. It has been reported in the literature that a few GH29 enzymes exhibit transfucosylation capacity and can be driven by different acceptor sugars (e.g., GlcNAc). This propensity is frequently associated with tolerance to pNP-derivatives and supports the rationale for their preference in engineered biocatalysis applications [21].

GH29 α-L-fucosidases from another intestinal bacteria, Bacteroides genus, generally exhibit optimum activity at near-neutral pH (~6.5–7.5) and around 37 °C. Among the GH29 α-L-fucosidases from Bacteroides, BF3242 from *B. fragilis* can synthesize Fuc-GlcNAc at near-neutral conditions (pH∼7.5, 37 °C) with high yields using pNP-Fuc as the donor and GlcNAc as the acceptor. This performance can be further improved by engineering (regioselectivity/transfucosylation) [22,23]. In contrast, the slightly acidic optimum (pH ∼5.5) and activity maintained over a moderate temperature range (24–42 °C) of *B. castoris* enzyme offer a complementary window to the neutral-pH-regulated Bacteroides enzymes, thus enabling flexible design of workflows for acidic processes, low-buffer food/culture media, and energy-efficient moderate temperatures. This difference suggests an application-based division of labor between the propensity of BF3242 for the synthesis of HMO-like structures in near-neutral conditions and the suitability of *B. castoris* enzyme for hydrolysis/pretreatment or acid-tolerant synthesis schemes in the acidic range [22,23]. Enterococcus-derived EntFuc (GH29) appears to reach its highest activity at neutral pH (~7.0) and low temperature (30 °C). This profile may be preferred in biosynthetic processes suited to low-temperature and neutral pH processes but appears less suitable for applications at more acidic and physiological temperatures [24]. The more acidic optimum and wider operating range (pH 3.0–8.0; 24–42 °C) of *B. castoris* enzyme expands the process window, thus allowing rational design of sequential/hybrid lines according to ambient pH, with EntFuc suitable for neutral conditions. Similarly, marine bacterial GH29 fucosidases such as *Paraglaciecola* sp. Fp231/Fp239/Fp284 have optimum activity around pH 5.6–6.0 but are active at low temperatures (~25 °C). This enzyme exhibits high linkage selectivity toward Fuc(α1,4)GlcNAc [25,26]. In contrast, the *B. castoris* enzyme provides operational flexibility in acidic mild conditions. Therefore, sequencing with Paraglaciecola in cold-activated/bond-specific steps and *B. castoris* in extensive hydrolysis/synthesis steps in acidic mild conditions significantly expands the process window. Similarly, although Alf1_Wf, the GH29-A α-L-fucosidase from *Wenyingzhuangia fucanilytica*, can hydrolyze some natural substrates and pNP-Fuc, the combination of an acidic optimum and a moderate temperature range in the *B. castoris* enzyme offers practical advantages by enabling operation under mildly acidic conditions and 24–42 °C, thereby broadening the biocatalytic process window and easing scale-up.

When assessed under identical assay conditions, the *B. castoris* enzyme demonstrated a marginally lower maximal activity than the well-characterized commercial α-L-fucosidase derived from *T. maritima*. Our comparative activity profiles indicate distinct stability characteristics between the two enzymes. *B. castoris* α-L-fucosidase consistently maintained high activity at pH 5.5, even when the reaction temperature varied within the 24–42 °C range, indicating that its performance is predominantly governed by pH rather than thermal fluctuations (Figure 5). This feature suggests that the enzyme is particularly well-suited for acidic milieus, such as gastrointestinal or lysosomal environments, where pH is the primary stress factor and it reflects the adaptation of *Bifidobacterium* to the fluctuating environment of the intestinal niche in which it naturally resides. Importantly, even though the enzyme’s activity declined outside this optimum, the profile indicated sufficient catalytic output within a moderately broad temperature window, supporting its applicability in conditions where precise temperature control may not be feasible. In contrast, *T. maritima* α-L-fucosidase displayed a complementary stability pattern. Its catalytic activity was sustained across a pH range of 3.0–8.0, highlighting a degree of pH tolerance. However, the enzyme’s performance was strongly dependent on temperature, with significant variation in activity upon thermal shifts. Such behavior is consistent with its thermophilic origin, where adaptation to high and stable temperatures is critical, but less selective pressure exists for pH specialization. Collectively, these findings emphasize the distinctive adaptive strategies of the two enzymes. While *T. maritima* fucosidase is optimized for thermophilic environments, the *B. castoris* enzyme demonstrates superior resilience under acidic conditions, underscoring its potential utility in applications requiring stability at low pH.

The activity of the *B. castoris* enzyme across the pH range of 3.0–8.0 and the temperature range of 24–42 °C, particularly its stability at 42 °C and pH 5.5, may provide advantages in both hydrolytic and synthetic applications. Functionally, GH29 enzymes are known for their ability to act on a wide range of α-linked fucose residues (e.g., α-1,2, α-1,3, α-1,4, α-1,6), with documented applications in transfucosylation reactions to synthesize fucosylated glycans [27]. *B. castoris* LMG 30937 also encodes an α-fucosidase of family GH95, as revealed by sequence similarity analyses [18]. The coexistence of GH29 and GH95 fucosidases within a single organism likely reflects complementary roles in the breakdown of fucose-containing oligosaccharides. Such redundancy may confer ecological advantages in the gut environment, particularly for hosts such as European beavers and wild mouse, in which *B. castoris* has been found to thrive. The broad substrate utilization profile of *B. castoris* described in recent genomic and ecological studies further emphasizes the significance of the enzyme reported here. The presence of a robust GH29 α-L-fucosidase supports the hypothesis that this species contributes to fucose degradation within the host gut, potentially influencing host–microbe interactions and shaping the gut glycome. These findings resonate with earlier studies that reported enriched glycosidase repertoires among bifidobacteria isolated from mammals, reinforcing their adaptation to complex carbohydrate-rich niches [28,29]. Another important feature is the recombinant yield and stability of the *B. castoris* enzyme. Recombinant expression often poses challenges, as seen with some Bifidobacterial glycosidases that suffer from low solubility or require elaborate refolding protocols. In our system, the *B. castoris* fucosidase was produced in a soluble and active form, underscoring its compatibility with heterologous expression (Figure 3). This ease of production enhances its potential for scalable applications, setting it apart from certain bifidobacterial enzymes that remain difficult to harness industrially [30].

Further research should be directed toward an in-depth characterization of the enzyme, encompassing kinetic parameter analysis (*Km, Vmax, kcat*) with physiologically relevant substrates, including fucosylated glycans, glycoproteins, and glycolipids. Comparative structural analysis through crystallography or advanced computational modeling may reveal the determinants of substrate specificity and guide enzyme engineering efforts aimed at enhancing catalytic efficiency or stability under industrial conditions. Additionally, exploring its activity in physiological matrices such as milk glycans, mucin-derived oligosaccharides could clarify its potential application in probiotic development, glycoengineering, and therapeutic glycan remodeling. In summary, this study provides the first experimental evidence for the activity of a GH29 α-L-fucosidase from *B. castoris*. Its near-physiological optima, activity stability across conditions, and alignment with known GH29 enzyme properties position it as a promising candidate for further exploration in both fundamental microbiology and applied biotechnology.

## 4. Materials and Methods

### 4.1. Materials

*B. castoris* LMG 30937 was obtained from Belgian Coordinated Collections of Micro-organisms (Ghent, Belgium). Luria Broth (110285), Man–Rogose–Sharp (110660), Anaerobic jar 2,5 L-volume (116387), Anaerocult^®^ A (113829) and Anaerotest^®^ (132371) were obtained from Merck Millipore (Darmstadt, Germany). Platinum™ II Hot-Start PCR Master Mix (2×) was obtained from Invitrogen (Leicestershire, United Kingdom). The Expresso™ Rhamnose Cloning and Expression Kit (49013-1) was purchased from Lucigen (Middleton, WI, United States of America). Qubit™ Protein Assay Kit (Q33211) was purchased from Thermo Fisher Scientific (Waltham, MA, United States of America). HisPur™ Ni-NTA Resin (25214) was purchased from Thermo Scientific (Rockford, IL, United States of America)). 4-Nitrophenyl α-L-fucopyranoside (sc-216987) was purchased from Santa Cruz Biotechnology (Dallas, TX, United States of America). *Thermotoga maritima*-derived α-L-fucosidase was purchased from Megazyme (Bray, Ireland). L-cysteine (168149), L-rhamnose monohydrate (R3875), kanamycin (K1377) and ampicillin (A9393) were obtained from Sigma-Aldrich Chemical Co. (St. Louis, MO, United States of America).

### 4.2. Methods

#### 4.2.1. Bacteria and Media

*B. castoris* was grown in Man–Rogose–Sharp (MRS) broth supplemented with 0.05% (*w*/*v*) L-cysteine. The cells were anaerobically grown in an anaerobic jar at 37 °C for 48 h, using Anaerotest A and Anaerocult A to maintain anaerobic conditions. The *E. cloni* 10G chemically competent cells were grown in Luria Broth (LB) media (agar or broth) containing kanamycin (33 µg/mL).

#### 4.2.2. In Silico Analysis

**Figure A1:**
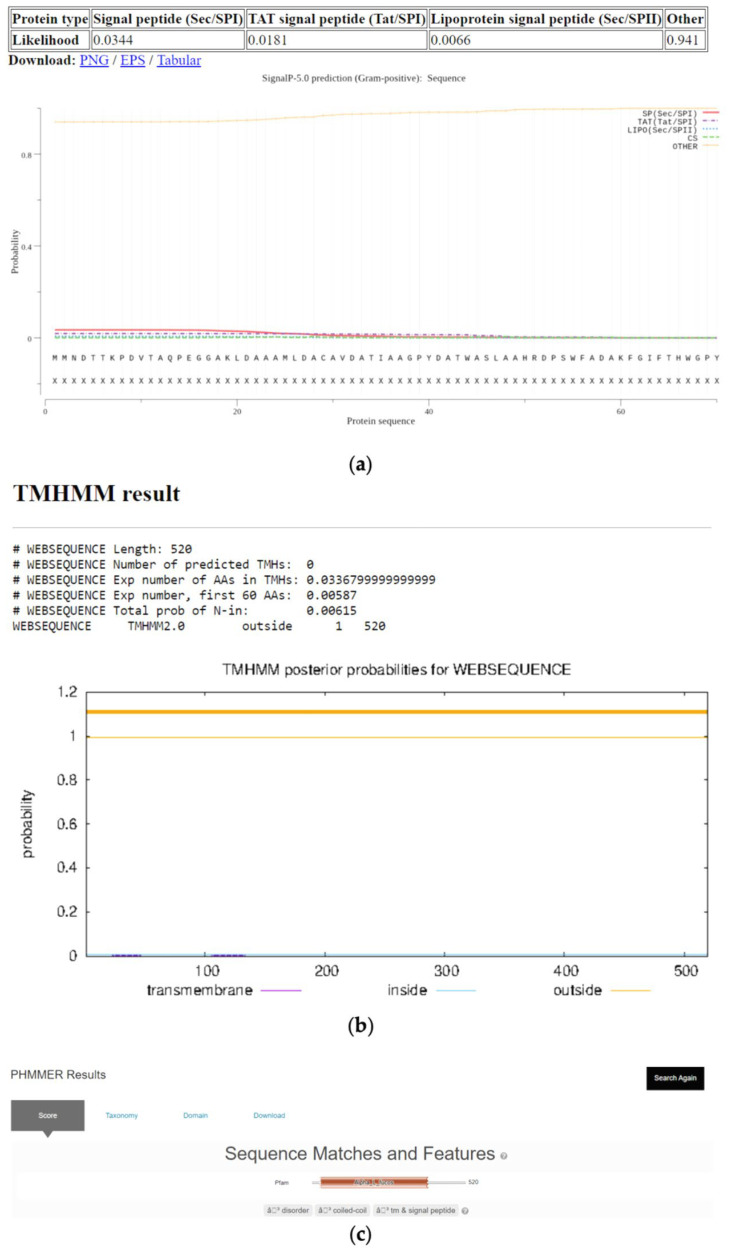
In silico prediction of signal peptides, transmembrane domains, and functional domains of the candidate enzymes: (**a**) SignalP 5.0, (**b**) TMHMM 2.0, (**c**) HMMER domain analysis.

**Figure A2:**
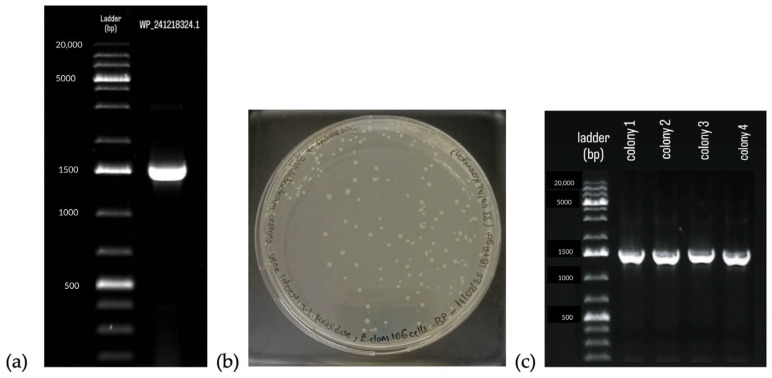
(**a**) Agarose gel electrophoresis of the PCR-amplified gene (WP_241218324.1; 1563 bp), (**b**) colony formation after transformation, (**c**) and colony PCR confirmation results during the recombinant production of the WP_241218324.1-coded enzyme using the N-His-SUMO-tagged pRham expression vector.

In silico analyses were conducted to identify potential signal peptides and transmembrane regions in the amino acid sequences of the candidate enzymes prior to recombinant production. SignalP 5.0 (accessed on 28 June 2024) and TMHMM 2.0 (accessed on 28 June 2024) servers were employed to predict signal peptides and transmembrane domains, respectively. According to SignalP predictions based on hidden Markov models and artificial neural networks, none of the enzymes showed significant signal peptide probabilities (≥0.4), and no cleavage sites were detected. Similarly, TMHMM analyses revealed no transmembrane helices with high probability scores (≥1.0), indicating that the enzymes are likely soluble proteins. These findings were further supported by HMMER-based domain analysis (accessed on 28 June 2024), which confirmed the absence of membrane-associated or secretion-related domains (Figure A1).

#### 4.2.3. Cloning, Expression and Purification α-L-Fucosidase

Expresso™ Rhamnose Cloning and Expression Kit (Lucigen, Middleton, WI, United States of America) was used for gene cloning. The α-L-fucosidase coding sequence (accession number WP_241218324.1) was amplified from *B. castoris* LMG 30937 genomic DNA with primers; 5′-CGCGAACAGATTGGAGGTAACGACACGACGAAACCGGAC-3′ and 5′-GTGGCGGCCGCTCTATTAGCCGACATGCAATCGGAAGCC-3′. PCR conditions were as follows: start at 95 °C for 5 min for 1 cycle to release of genomic DNA (first denaturation), followed by 40 cycles of 95 °C for 30 s (initial denaturation), 60 °C for 30 s (annealing step) and 72 °C for 1 min (elongation step), finally 1 cycle of 72 °C for 10 min (final extension step). PCR product containing an N-terminal 6xHis-SUMO tag was examined by agarose gel electrophoresis. PCR product and the pRham™ N-His SUMO vector was transformed into *E. cloni* 10G chemically competent cells for protein expression. The sequence was confirmed (SUMO Forward: 5′–ATTCAAGCTGATCAGACCCCTGAA–3 and pETite Reverse: 5′–CTCAAGACCCGTTTAGAGGC–3′). The transformed cells were screened by using the LB agar plate containing kanamycin (33 ug/mL) at 37 °C for 24 h and confirmed target gene region into vector using colony PCR using sequenced primer which provided by Expresso™ Rhamnose Cloning and Expression Kit. DNA fragment with the expected molecular size was sequenced for final confirmation. Successful transformants were cultivated in LB supplemented with 33 µg/mL antibiotic at 37 °C until the optical density at OD_600_ reached approximately 0.5, after which protein expression was induced by the addition of L-rhamnose to a final concentration of 2% and incubation at 24 °C for 16 h.

The protein was purified following bacterial lysis using a batch method with HisPur™ Ni-NTA Resin pre-equilibrated with equilibration buffer (20 mM NaH_2_PO_4_, 300 mM NaCl, 10 mM imidazole). The resin-bound protein was washed with wash buffer (20 mM NaH_2_PO_4_, 300 mM NaCl, 25 mM imidazole) to remove potential contaminants and subsequently eluted with elution buffer (20 mM NaH_2_PO_4_, 300 mM NaCl, 250 mM imidazole). The enzyme concentration was determined using the Qubit™ Protein Assay Kit. Purified samples were analyzed by SDS-PAGE using a 4–12% discontinuous gel system and visualized with Coomassie Brilliant Blue staining [31].

#### 4.2.4. Enzyme Activity Measurement and Determination of Optimum Reaction Conditions

The α-L-fucosidase activity assay and the determination of optimum reaction conditions were developed by integrating elements from previously established methods with several modifications [26,31]. Based on these refinements, the assay was performed using 5 mM pNP-α-L-fucopyranoside (pNP-fucose) as a substrate in 100 μL of 50 mM sodium acetate buffer (pH 5.5), followed by incubation at 37 °C for 60 min with shaking at 200 rpm. Reactions were terminated by addition of an equal volume of 10% Na_2_CO_3_, and absorbance was measured at 405 nm. The optimal pH was determined using 50 mM Na-acetate buffer for pH 3.0, 4.5 and 5.5 and 50 mM Tris-HCl buffer for pH 7.0 and 8.0, 1 μg novel α-L-fucosidase and 2 mM pNP-fucose. Optimal temperature was determined in 50 μL reaction volume between 24 °C, 37 °C and 42 °C. After a 1 h incubation at 37 °C and 200 rpm, the reaction was terminated by the addition of an equal volume of %10 Na_2_CO_3_ buffer. 1 μg of *Thermotoga maritima* (*T. maritima*) -derived α-L-fucosidase was used as a positive control under the same conditions. The absorbance was measured at 405 nm. All the assays were performed in triplicate.

Due to the basic environment (pH > 9.0) provided by the Na_2_CO_3_ buffer, the released p-nitrophenol is ionized and converted into p-nitrophenolate (pNP^−^) form. The concentration of pNP^−^ ion was calculated using a path length of 0.5 cm and the molar absorption coefficient of ε = 1.75 × 10^4^ M^−1^ cm^−1^ from the Lambert-Beer law. One unit of enzyme activity (U) was defined as the amount of enzyme that formed 1 μmol of p-nitrophenolate ion per minute. Specific activity was expressed as the ratio of unit enzyme activity to the amount of protein (U/mg protein) [32]. The activity profiles of both the novel and commercial α-L-fucosidases across different pH values and temperatures were visualized using NCSS 2025 (NCSS LLC, Kaysville, UT, United States of America) program. Line graphs with error bars (mean ± SD) were generated to illustrate the effect of pH and temperature on enzyme activity, while three-dimensional surface plots were created to depict the overall activity landscape and to identify optimal reaction conditions.

#### 4.2.5. Statistical Data Analysis

In the study, NCSS 2025 (NCSS LLC, Kaysville, UT, United States of America) program was used for statistical analysis. First, to evaluate the effects of pH (5 levels: 3.0 to 8.0) and temperature (3 levels: 24 °C, 37 °C, and 42 °C) on the enzymatic activity of the novel α-L-fucosidase, a two-way ANOVA was performed, considering pH and temperature as factors. Interaction effects between the two variables were also examined. When statistically significant differences were detected, Tukey’s multiple comparison test was applied as a post hoc analysis to identify specific group differences.

## 5. Conclusions

This study presents the first experimental characterization of a GH29 α-L-fucosidase from *B. castoris* LMG 30937, thereby broadening the current understanding of bifidobacterial glycosidases and their ecological functions within mammalian gut environments. Although the gene encoding this GH29 α-L-fucosidase was previously annotated in genomic databases, no experimental confirmation of its enzymatic activity was available prior to this study. Our study provides the first biochemical evidence confirming the predicted function, thus laying the groundwork for subsequent investigations into the enzyme’s structural properties, substrate scope, and potential physiological significance. In this context, the designation of the enzyme as “novel” reflects not only its recent discovery in *B. castoris* but also its transition from a computational prediction to an experimentally characterized biocatalyst. The enzyme was successfully produced in a soluble and active recombinant form, showing distinct pH and temperature-dependent activity with optimal performance at pH 5.5 and 42 °C. Its stability across acidic conditions and moderate temperature ranges underscores its adaptation to intestinal niches and highlights functional differences from thermophilic enzymes such as *T. maritima* α-L-fucosidase. The stability of the *B. castoris* enzyme under acidic conditions and its activity at pH 3.0–8.0 and temperature range of 24–42 °C make it a valuable candidate for glycan hydrolysis, trans-fucosylation, and therapeutic or industrial glycoengineering applications. Comparatively, the enzyme’s strong activity against pNP-fucoside is consistent with GH29-A-like behavior and distinguishes it from GH29-B members such as *B. bifidum* AfcB, while its acidic optimum and intermediate temperature range are complementary to the nearly neutral GH29s from Bacteroides and cold-active, vineyard-specific marine enzymes, and the thermophilic *T. maritima* α-L-fucosidase, whose performance is primarily temperature-dependent. Furthermore, its ease of heterologous production enhances its applicability for scalable use. Collectively, these findings establish a foundation for future structural, kinetic, and functional studies to explore the enzyme’s biological relevance and biotechnological potential.

## Figures and Tables

**Table 1:** Enzyme activity (mean ± SD, U/mg) at each pH level under three temperatures (24 °C, 37 °C, 42 °C). Tukey–Kramer adjusted *p*-values indicate the significance of difference between that pH and the optimal pH 5.5 at the same temperature (- not applicable for the reference condition).

Temperature (°C)	pH	Mean Activity ± SD (U/mg)	Tukey *p*-Value
24	3.0	0.001 ± 0.004	<0.001
24	4.5	0.033 ± 0.004	<0.001
24	5.5	0.189 ± 0.004	-
24	7.0	0.034 ± 0.004	<0.001
24	8.0	0.006 ± 0.004	<0.001
37	3.0	0.001 ± 0.004	<0.001
37	4.5	0.038 ± 0.004	<0.001
37	5.5	0.242 ± 0.004	-
37	7.0	0.064 ± 0.004	<0.001
37	8.0	0.009 ± 0.004	<0.001
42	3.0	0.001 ± 0.004	<0.001
42	4.5	0.022 ± 0.004	<0.001
42	5.5	0.264 ± 0.004	-
42	7.0	0.084 ± 0.004	<0.001
42	8.0	0.009 ± 0.004	<0.001

## Data Availability

The original contributions presented in the study are included in the article; further inquiries can be directed to the corresponding author.
